# Quorum sensing LuxS/autoinducer-2 inhibits *Enterococcus faecalis* biofilm formation ability

**DOI:** 10.1590/1678-7757-2017-0566

**Published:** 2018-10-02

**Authors:** Yue Yang, Wenzhi Li, Benxiang Hou, Chen Zhang

**Affiliations:** 1 Capital Medical University School of Stomatology, Department of Endodontics, Beijing, China

**Keywords:** Biofilm, Enterococcus faecalis, Quorum sensing

## Abstract

**Objective:**

To investigate the relation between biofilm formation ability and quorum sensing gene LuxS/AI-2.

**Materials and Methods:**

*Enterococcus faecalis (E. faecalis)* standard strain ATCC 29212 was used in the study. Long flanking homology polymerase chain reaction method was used to build the LuxS gene knockout strain. Sequential culture turbidity measurement and CFU counting were used to assess the proliferation ability of *E. faecalis* after the depletion of LuxS. 96-well plate assay was used to quantify the biofilm formation ability; CLSM was used to observe the attached bacteria areas, while scanning electron microscopy (SEM) was performed to observe biofilm microstructure conditions.

**Results:**

LuxS gene knockout strains were successfully constructed and identified. The results showed that proliferation ability of *E. faecalis* was not affected by the depletion of the luxS gene, and the biofilm formation ability of ΔLuxS 29212 significantly decreased (P<0.05).

**Conclusions:**

Collectively, our studies provide the LuxS gene's key role in controlling biofilm formation of *E. faecalis,* which presented a negative regulation, and furthermore, providing us a possible way to conquer the persistent apical periodontitis.

## Introduction

Approximately 10-20% of teeth are not healed by apical lesions after re-treatment in endodontic therapy; this condition is called persistent apical periodontitis (PAP). Bacterial infection was recognized as the dominant cause of PAP ^1^ . The survival of bacteria goes hand in hand with their ability to live in an adverse environment, and biofilm formation provides them a defensive barrier against a hostile situation ^2^ . A number of investigations have shown that most root canal treatment failures are caused by microorganisms surviving around the apical foramen by forming a biofilm structure, invading the extraradicular area, and attaching to the cementum around the root apex ^3^ ^-^ ^6^ . The eradication of apical microorganisms by endodontic microsurgery resulted in a successful outcome based on a variety of follow-up studies ^7^ ^-^ ^9^ .

Biofilm formation is considered as the primary pathogenic factor resulting in persistent infections and treatment failures, while *Enterococcus faecalis (E. faecalis)* has been reported to be a highly detected pathogenic bacteria from the extraradicular biofilm and is one of the highly investigated bacteria associated with PAP lesions ^10^ ^-^ ^13^ . However, the mechanism of the *E. faecalis* biofilm formation remains elusive.

During bacterial amplification, many signaling molecules are released and allow for cell-to-cell communication. Once these molecules reach a threshold level, bacterial behavior will change in ways, including extracellular matrix production, bacterial surface adhesion and virulence factor expression ^14^ . Quorum sensing (QS) is an important mechanism that controls the signal delivery of microorganisms and plays an important role in regulating gene expression and biofilm formation. The signal delivery of most Gram-negative and -positive microorganisms relies on the LuxS/autoinducer-2(AI-2) quorum sensing system ^15^ ^-^ ^17^ . Recently, the LuxS gene-mediated quorum sensing (AI-2 signaling) was found to plays an important role in interspecies communication and has been involved in bacterial virulence, persistence infections and biofilms formation in bacteria, such as *Streptococcus mutans, Escherichia coli,* and *Actinobacillus* . ^14^ ^,^ ^18^ ^,^ ^19^ Nevertheless, the relation between the LuxS gene expression levels and the biofilm formation ability of the pathogenic bacteria *E. faecalis* remains unknown.

To detect the relation between LuxS gene expression levels and the biofilm formation ability of *E. faecalis* and to determine whether the LuxS gene plays a crucial role in the pathogenicity of *E. faecalis,* a series of experiments was conducted. We established a LuxS gene knockout strain and investigated the function of the LuxS gene in the biofilm formation process directly by comparing the biofilm formation ability between the gene knockout strain and the ATCC standard strain. The study provides us a favorable way to understand the mechanism of infection and a new direction of treatment method. A thorough knowledge of the LuxS/AI-2 signaling system and apical biofilm formation could guide new strategies to combat infections, leading to a better prognosis for root-canal re-treatments.

## Materials and methods

### Bacteria culture

A brain-heart infusion (BHI) broth supplemented with 5 g yeast extract/L and 5% v/v vitamin K+ hemin (BHI-YE; Becton, Dickinson and Company, Franklin Lakes, NJ, USA) was used to grow the bacteria. Bacterial strains were grown at 37°C in an anaerobic environment, using gas-generating sachets (Gas-Pak EZ; Becton, Dickinson and Company, Franklin Lakes, NJ, USA) to produce the required environment.

### Construction of the *E. faecalis* LuxS mutant strain

**Figure 1:**
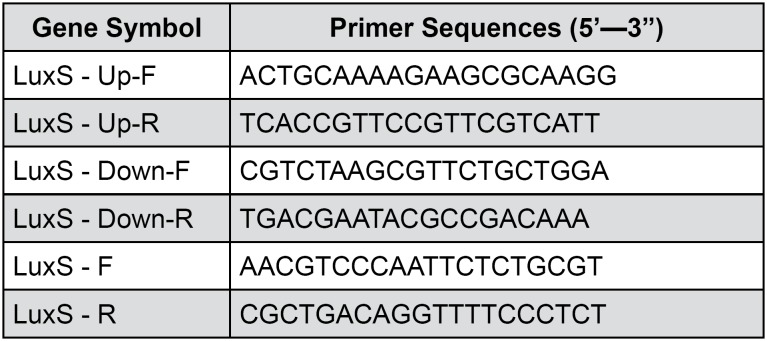
Primers sequences used in the real-time RT-PCR

Long flanking homology (LFH) PCR was used to generate deletion mutations, in which the designated coding region was largely replaced with an antibiotic resistance cassette as previously described. Strain ATCC 29212 chromosomal DNA was used for PCR amplification of flanking fragments of each gene using primers for the upstream and downstream of the LuxS gene. The PCR products were joined to a P-EASY vector to establish the plasmid termed as pEASY-LuxS-up-amp-kana-LuxS-down. The plasmid was selected using LB agar plate with ampicillin and kanamycin (50 ug/ ml). The successful plasmid construct was transformed into *E. faecalis* by electroporation according to the protocol. The LuxS gene knockout *E. faecalis* was identified by PCR using primers P1 to P4. The primers were listed in Figure 1 .

### Proliferation ability assessment using sequential culture turbidity measurements and CFU counting

Both strains were cultured under steady state, and the growth ability was investigated using sequential culture turbidity measurements (absorbance at 600 nm) ^20^ . The bacterial colony was harvested by sonication (Vibra Cell, Sonics & Materials Inc., Newtown, Connecticut, USA) for 2 min with 1-s pulses and 1-s gaps, both of which were simultaneously assessed over time. For the growth assay, 200-μl cultures were prepared in 96-well plates (SARSTEDT AG & Co, Rommelsdorfer, Nümbrecht, Germany) by adding 2 μl bacterial culture to 198 μl culture medium (i.e., diluted 1/100), and then were sealed. The plates were incubated at 37°C with shaking at 200 rpm (ThermoFisher Scientific, Waltham, Massachusetts, Australia). Readings of culture turbidity were acquired every 2 h from 0 h to 72 h using a plate reader (POLARstar Omega, Oldenburg, Germany). For CFU counts, 2 μl of the bacterial suspension was diluted 1:10000 into fresh BHI medium and plated on BHI culture medium plates, and CFU values were calculated after the plates were incubated anaerobically at 37°C for 72 h.

### Biofilm formation ability assessment using the 96-well plate assay

Briefly, the *E. faecalis* strain and mutant strain were grown overnight in BHI at 37°C. After 24 h of incubation at 37°C, wells were gently washed three times with 200 μL of PBS, dried in an inverted position, and stained with 0.5% crystal violet (pre-filtered through a 0.44 μm filter) for 1 min, rinsing the residual dye left only the stained cells attached to the surface of the wells. The crystal violet was solubilized in 100 μL of 95% ethanol (v/v), and OD570 readings were taken for quantification. Each assay was performed in triplicate and repeated three times.

### Confocal laser scanning microscopy (CLSM)

CLSM (FV300, Olympus, Tokyo, Japan) was performed to observe the attached bacterial areas. A sterile extracted root was inserted into the bacterial solution at a concentration of 10^^8^ to establish the biofilm formation model on the apical part *in vitro.* Three samples were selected and stained with the LIVE/DEAD® BacLight™ Bacterial Viability Kit solution (Molecular Probes, Inc., Eugene, OR, USA) according to the manufacturer's instructions. An argon laser (485±14 nm) was used as the excitation source for the reagents. Emitted fluorescent light was collected in 2 separate emission filters at 500 nm (SYTO 9; green-fluorescent nucleic acid stain) and 635 nm (propidium iodide; red-fluorescent nucleic acid stain). The collected images were analyzed by an image-processing program (FluoView 5.0, Olympus, Tokyo, Japan) to count the biofilm areas.

### Scanning electron microscopy (SEM)

Scanning electron microscopy (SEM) was performed to observe biofilm microstructure conditions. Three biofilm formation models were selected. The samples with biofilm formation were fixed in a 4% glutaraldehyde and paraformaldehyde solution in 0.1 M cacodylate buffer (pH 7.4) for 3 hours. Then, the fixed samples were washed 3 times with a 0.1 M cacodylate buffer for 10 minutes and dehydrated for 30 minutes in a graded series of ethanol. For SEM observation, ethanol was replaced by isoamyl acetate, and after reaching the critical point of drying, the samples were mounted on a stub and coated with gold. The biofilm surfaces were observed with variable-pressure field emission SEM (SUPRA55VP, Carl Zeiss, Oberkochen, Germany).

### Statistical methods

The statistical analyses were performed using SPSS 18.0 (Superior Performing Software Systems, Chicago, USA). All experiments were performed in triplicate. One-way ANOVA was used to conduct the statistical analysis; p values <0.05 were considered significant.

## Results

### Construction of *E. faecalis* LuxS mutants

#### Amplification of the up and down stream sequence of the LuxS gene

**Figure 2:**
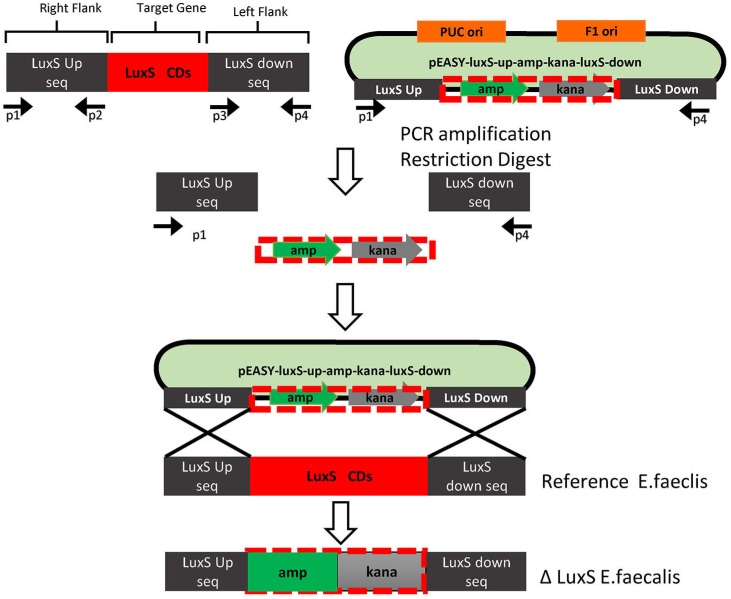
Overall strategy of PCR ligation mutagenesis The mutant strain was made as follows: 1. Construction of recombinant plasmids. Two pairs of gene-specific primers were designed. P1 and P2 are used to amplify the 5′ upper stream homologous arm of LuxS gene, while P3 and P4 amplify the 3′ region flanking the target gene. Each DNA fragment was ligated to the VECTOR according to the manufacturer′s instruction. The recombinant plasmid was then transformed into E. coli DH5a competent cells. Positive clones of transformed cells were selected and sequenced, named as pEASYLuxS-up-amp-kana-LuxS-down. Under these conditions, P2 has an expanded HindIII site, whereas P3 has an expanded XhoI site, both attached to their 5′ ends. 2. Transformation of *E. faecalis* . *E. faecalis* was transformed by electroporation according to the protocol. The E. faecalis LuxS gene knockout was identified by PCR with primers P1 to P4

As first step to construct LuxS mutant *E. faecalis,* the upstream and downstream regions of the LuxS gene in *E. faecalis* were amplified. The products of flanking regions are 961 bp and 820 bp, respectively ( Figure 2 ).

#### Construction of recombinant plasmid.

**Figure 3:**
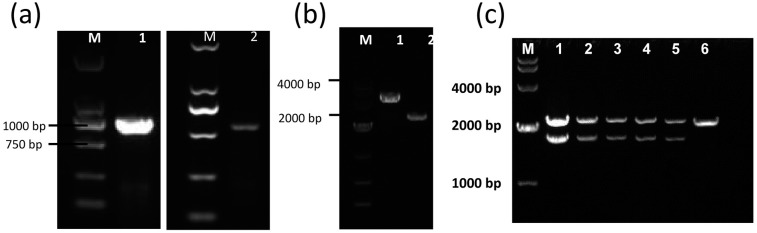
The deletion of LuxS gene did not affect the proliferation ability of *E. faecalis.* (a) The growth curves of Δ LuxS *E. faecalis* strain and the ATCC 29212 strain every 2 h from 0 h to 72 h. (b) The CFU counting of Δ LuxS *E. faecalis* strain and the ATCC 29212 strain

The sequence of the LuxS gene upstream and downstream was inserted into the pEASY-Y vector, and the plasmid pEASY-LuxS-up-amp-kana-LuxS-down was established. The length of the vector successfully introduced with up and down stream sequence product has approximately 3000 bp ( Figure 3a, b ).

### Identification of LuxS gene knockout *E. faecalis*

In the wild type *E. faecalis,* the amplified product uses primers p1 and p4 that will result in a 1500 bp product, whereas re-constructed plasmid with primers p1 to p4 will result in a PCR product of approximately 2500 bp. We chose 6 clones after electroporation, and only detected re-constructed plasmid in the 6 band, which indicates that the LuxS gene in *E. faecalis* genome was deleted ( Figure 3c ).

### ΔLuxS *E. faecalis* proliferation ability is not affected by gene knockout

**Figure 4:**
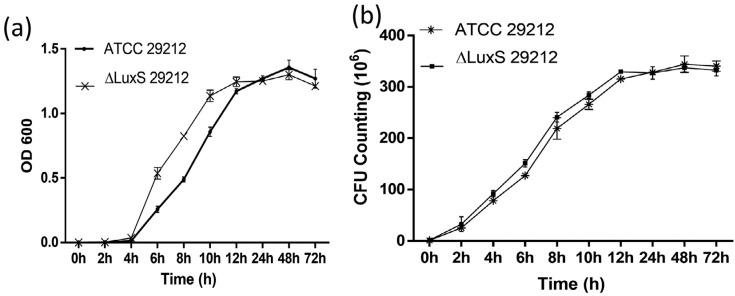
Building the *E. faecalis* LuxS deficient strain ΔLuxS 29212 by LFH-PCR Amplification of the upper and down-stream homologous arm of the E. faecalis LuxS gene. M: Marker DL2000 1: LuxS upstream homologous arm fragment (P1 to P2), length is 961 bp; 2: LuxS down-stream homologous arm fragment (P3 to P4), length is 820 bp; (b) The identification of recombinant plasmid. M: Marker 1: Recombinant plasmid 2: pEASY-T1 vector; (c) The identification of the LuxS gene knockout in *E. faecalis* . M: DNA markerDGL 4000; Lane 1-5, upper band: recombination vector LuxS up and down homologous arm fragment interpose with the amp and kana genes; lower band: LuxS gene from up to low homologous arm (P1 to P4)

To compare the bacteria growth between the standard strain and the gene knockout strain, we cultured both strains under steady state and measured the optical density value (OD600) which mirrors the growth ability of bacteria. The result showed that after 4 hours of culture both strains reached the logarithmic phase, and the OD600 values increased along with culture time. From 4 h to 12 h, the OD600 value of the mutant strain is larger than the standard strain (P>0.05); after 12 h incubation, both strains reached platform phase ( Figure 4a ). No difference was found in the CFU counts between the two strains ( Figure 4b ).

### LuxS gene depletion significantly decreased the biofilm formation ability of *E. faecalis*

**Figure 5:**
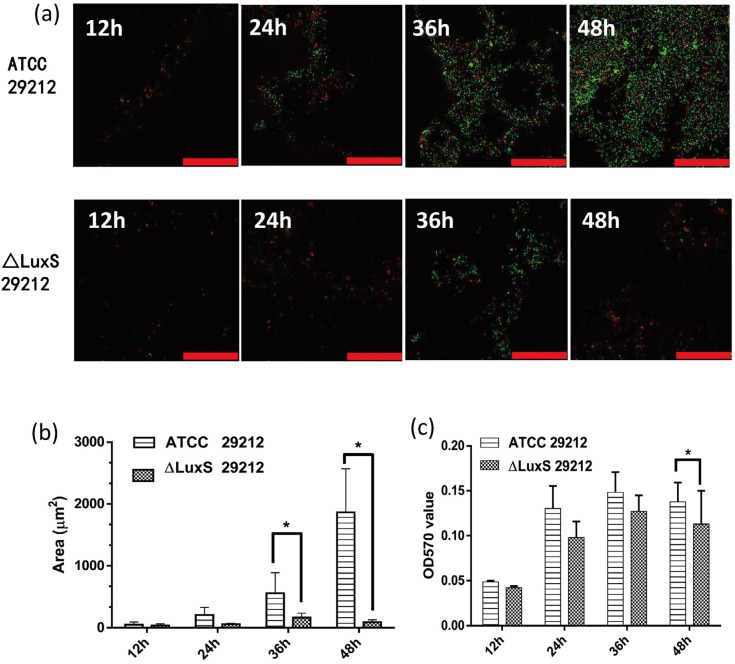
The deletion of LuxS gene decreased the biofilm formation ability of *E. faecalis.* (a) Confocal laser scanning microscope images of biofilm formation on Δ LuxS *E. faecalis* strain at 12 h, 24 h, 36 h and 48 h. Scale bar=50 μm; (b) The biofilm areas were measured using an image-processing program; (c) Quantifying the biofilm by crystal violet staining, One-way ANOVA was used to analyze the statistical significance. All error bars signify standard deviation (n=3). *:P<0.05

**Figure 6:**
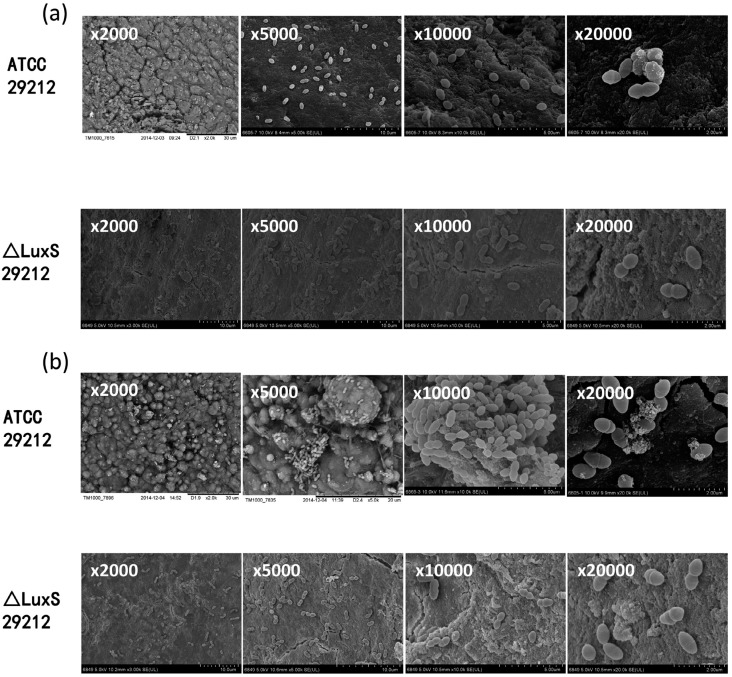
Scanning electron microscopic images of biofilm formation in the Δ LuxS *E. faecalis* strain. The microstructure of the biofilm was detected at 12 h (a) and 48 h (b) by a scanning electron microscope (SEM) under the magnifications: x2,000, x5,000, x10,000, and x20,000

To investigate the biofilm formation ability after the deletion of the LuxS gene, biofilm formation and areas were detected via the biofilm CLSM, and the biofilm biomass was measured by CV staining at 12 h, 24 h, 36 h and 48 h, while the biofilm microstructure conditions were observed by SEM. The results of the CLSM demonstrated that the biofilm areas increased from 12 h to 48 h in both strains. A large sheet biofilm was observed in standard strains at 36 h and 48 h, while there was only small and dispersive biofilm formation in ΔLuxS 29212, and its biofilm formation at 48 h was even looser than 36 h ( Figure 5a ). Meanwhile, the biofilm areas in standard strains were significantly larger than the mutant strains ( Figure 5b ). The result of CV staining showed that the biofilm biomass of both strains increased from 12 h to 48 h, while compared with standard strains, the biofilm biomass of ΔLuxS 29212 decreases significantly at 48 h ( Figure 5b ). Finally, the SEM images under the magnifications x2,000, x5,000, x10,000, x20,000 showed a large number of bacterial attached to the root surface at 12 h in standard strains, while there were only a few attached to ΔLuxS 29212 ( Figure 6a ). The images at 48 h showed that masses of bacteria were layered on the surface of the apical tissue; grainy secretions and filaceous links among bacteria were observed in standard strains while a loose biofilm was observed in ΔLuxS 29212, and no secretions were found ( Figure 6b ). Collectively, these results indicated that the deletion of the LuxS gene significantly decreased the biofilm formation ability of *E. faecalis.*

## Discussion

Biofilm formation has been shown to be involved in a variety of microbial infections, which are the result of microbial interactions. Bacterial interactions and the signals from the environment play an essential part during this procedure. Through recruiting diverse bacterial species to the site of infection and forming biofilm structures, the bacteria display an effective defense system against the host's immune defenses to cause several persistent diseases, such as PAP. The QS system is wildly concerned with bacterial communications, biofilm formation and maturation. AI-2 is a major signaling molecule produced by QS system and is extensively detected among Gram-positive and Gram-negative bacteria, serving as a widespread language for interspecies communication ^21^ ^-^ ^23^ . In addition, the synthesis of AI-2 relays on the LuxS gene. In oral studies, the LuxS gene in *Streptococcus mutans,* which is related to cariology, has been previously clarified ^19^ , but the role of the LuxS gene in *E. faecalis,* the main pathogen of PAP, still has not been elucidated. To investigate the LuxS gene function, we built a LuxS gene knockout strain and detected the role of LuxS gene in biofilm formation directly by comparing its biofilm formation ability with the standard strain.

Knockout technology is an invaluable experimental tool for investigating functions to genes, it provides tremendous perception into understanding the disease process. Key elements of this approach are the precise targeting of the gene and complete replacement of all copies of the gene in the genome. To detect the direct involvement of the LuxS gene, we established a LuxS mutant *E. faecalis* strain. The gene knockout enabled to test the specific function of the LuxS gene and observe the biofilm formation change regulated by quorum sensing system.

The aggregation of bacteria developed into a biofilm on a solid surface and produced extracellular polysaccharide substances, thus it constitutes a physical protection barrier and enhances their survivability and pathogenicity. The formation of biofilm relies not only on the extracellular matrix produced, but also on the large quantity of proliferated bacteria. Previous studies on oral bacteria found that different AI-2 concentrations could affect the biovolume, average biofilm thickness, and the architecture of biofilm formation, ^24^ which indicated that alteration of AI-2 expression caused by the knockout of LuxS may affect the growth of bacteria. However, in our study, we found that the proliferation ability of *E. faecalis* was not affected by the gene knockout, which provided the basis for the further experiments.

We measured the biofilm formation ability of *E. faecalis* strains quantitatively and qualitatively through 96-well plate assay, confocal laser scanning microscopy and scanning electron microscope. The microtiter plate (also called 96-well plate) assay for studying biofilm formation is a method which allows for the observation of bacterial adherence to an abiotic surface. In this assay, bacteria are incubated in vinyl “U”-bottom or other types of 96-well microtiter plates. Following the incubation period, planktonic bacteria are rinsed away, and the remaining adherent bacteria (biofilms) are stained with crystal violet dye, thus allowing visualization and quantization of the biofilm. We found that the biofilm formation ability significantly decreased in LuxS depletion strain compared with the standard strain ATCC 29212. CLSM images showed a small, dispersive and loose biofilm formation in ΔLuxS 29212, while the SEM showed a loose biofilm with absence of secretions and filaceous links in it. Above all, the LuxS gene may be involved in the attachment and aggregation of bacteria during the biofilm formation and may be the key gene that controls biofilm formation of *E. faecalis.* Previous studies by other authors revealed that LuxS/AI-2 could down-regulate metabolism-related enzymes of *E. faecalis* through proteomics analysis and speculated AI-2 signaling plays an important role in the regulation of a number of important metabolic properties of *E. faecalis* and even played essential roles in other oral bacteria ^25^ , such as *Streptococcus mutans, Escherichia coli, Porphyromonas* and *Actinobacillus* ^14^ ^,^ ^18^ ^,^ ^19^ ^,^ ^22^ ; these results are consistent with our study.

## Conclusion

In conclusion, LuxS gene depletion broke the biofilm formation process of *E. faecalis,* which demonstrated that the LuxS gene has an important role in the biofilm formation process of *E. faecalis* and provided us a new nonsurgical strategy to treat persistent apical periodontitis. However, further studies are still needed to clarify the regulatory mechanism of the LuxS/AI-2 QS system on *E. faecalis* biofilm formation.
